# Device Pocket Challenges in Elderly and Thin Individuals

**DOI:** 10.7759/cureus.12902

**Published:** 2021-01-25

**Authors:** Ramil Goel

**Affiliations:** 1 Cardiovascular Disease, University of Florida, Gainesville, USA

**Keywords:** thin patients, pocket reinforcement, pacemaker, implantable cardioverter defibrillator, deficient soft tissue

## Abstract

Aging results in loss of subcutaneous body fat as well as lean body mass. Elderly patients are also more likely to require cardiac implantable electronic devices (CIED) due to rising cardiovascular disease prevalence. A majority of the currently available devices require placement in a pocket created in the subcutaneous space between the subcutaneous fat tissue and the underlying chest wall muscle. Deficient subcutaneous fat tissue can result in device protrusion and even erosion through the skin. This can lead to significant morbidity and mortality especially when associated with device infection and need for device system extraction.

This article reviews the scope of the problem and some of the strategies that can be employed to address the lack of subcutaneous soft tissue at the time of device implant.

## Introduction and background

The world population is aging and the proportion of individuals above 65 years of age is projected to increase from 6.9% to 12% between 2000-2030 [1]. Aging is associated with increased risk of cardiovascular disorders and hence a need for cardiac implantable electronic devices (CIED) [2].

Aging is also associated with body fat redistribution and a general loss of subcutaneous fat [3]. An increase in visceral adipose tissue is accompanied by a loss in subcutaneous fat tissue [4]. 

Thin-skinned patients are at risk for progressive erosion from the CIED components, including the can and the leads. The lack of fat cushion leads to constant pressure by the device components directly against the skin. This can lead to gradual thinning of the skin, followed by eventual erosion and externalization of the device. While the initial results affect the cosmetic appearance and patient comfort, the externalization of the device implies system infection. The only effective way to treat this latter complication is device and lead extraction with its significant associated morbidity and mortality. The short-term 30-day mortality rate after lead extraction is around 2.1%, while the 1-year mortality rate is almost 8.4% [5]. The increased healthcare costs for admission, investigation, surgery, and new device implants also add to the burden of such complications.

A significant number of the device erosion-related complications in thin individuals can be avoided with awareness and relatively simple interventions to decrease the risk of device component pressure against the skin. Creating enough room in the pocket to prevent device protrusion towards the incision and skin is helpful, as is maintaining meticulous hemostasis to prevent excess pressure build-up in the pocket, which can also stretch the overlying incision and skin. However, these simple measures are often not adequate and other interventions are required for maintaining pocket integrity. These are discussed below.

## Review

Submuscular device implant

The placement of the CIED generator in a submuscular pocket deeper than the usual subcutaneous pocket is an effective technique to achieve an aesthetically effective device pocket without any prominent bulges or protrusion. The presence of another layer of soft tissue in the form of chest wall muscle also decreases the risk of device impingement on the skin leading to skin erosion. Most of these techniques require placing the device beneath the pectoralis major muscle. The different techniques involved are basically different approaches to access the sub-pectoral space. The anterior approach is perhaps the most widely used and involves separating the fibers of the sternal and clavicular heads of the pectoralis major muscle and accessing the sub-pectoral space through this separation. The lateral approach, separating the deltoid and pectoralis major muscle along the deltopectoral groove in order to access the sub-pectoral space, is another option. The anterior axillary approach involves making a lateral incision behind the anterior axillary fold and accessing the space beneath the pectoralis major muscle by creating a plane of dissection between the pectoralis major and minor muscles. The vascular access in this last approach is obtained from the infraclavicular incision and the leads are then tunneled through this subcutaneous space to the sub-pectoral pocket created through the axillary approach. These techniques require a good understanding of the superficial and deep regional chest wall regional anatomy [6]. Placement of subcutaneous only device is usually done beneath the latissimus dorsi muscle and is another example of submuscular device implant.

The submuscular techniques require the implanting proceduralist to have familiarity with local anatomical landmarks and competence with surgical skills. Due to the extensive dissection involved, the manipulation associated with these procedures, and the greater length of these procedures, general anesthesia is usually the best option for sedation strategy during these procedures. Subsequent device generator changes, lead revisions and upgrades requiring access to the pocket also become more challenging due to the need to dissect down to the submuscular plane. Pocket access, hemostasis, and closure are all steps that take up more time and operator experience. Although submuscular implantation of the device should decrease device erosion burden based on conventional wisdom, there is no actual data to look at the incidence of pocket erosion with this technique.

Leadless pacemaker

For almost 60 years since its invention and implantation, the permanent pacemaker required a separate pulse generator placed in the chest wall (initially occasionally in the abdominal wall) and a set of leads placed through the venous system, to provide portable pacing for ambulatory patients. The leadless pacemaker has been added to the armamentarium of cardiac electrophysiology recently, with FDA approving the Medtronic Micra device in 2016 [7]. This device, due to its innovative miniature design, has the pulse generator, battery, and pacing tines all placed in a compact capsule that can be implanted directly on the right ventricular myocardial surface. This obviates the need for a chest wall pocket to house a separate pulse generator. This may be an option for patients with deficient chest wall soft tissue.

The major limitation is, of course, the ability to pace only the ventricle, even though the latest version of the leadless pacemaker has an indirect mechanism to sense and track atrial contractions [8]. However, it is likely only a matter of time before the leadless pacing technology evolves further to provide effective multichamber pacing.

Soft tissue and skin reinforcement with acellular dermal matrix

Acellular collagen matrix consisting of a collagen and elastic fiber scaffold creates an additional layer of soft tissue between the device and the skin. This matrix is immunologically inert due to the removal of cellular content but it forms a soft layer of tissue reinforcing the pocket while forming the scaffold for host fibroblast cells to lay down an ingrowth of native tissue with revascularization and remodeling. The results in a case series of 24 elderly patients, with thin subcutaneous covering over their pockets, treated with matrix reinforcement was very encouraging [9]. This case series followed 24 patients with a mean age of 80 years, 14 with pacemakers and 10 with implantable cardioverter defibrillators (ICDs), who were deemed to be at risk for immediate skin breakdown due to thin soft tissue over their devices. These patients had pocket revision performed with a thick matrix patch placed beneath the skin covering the device and leads. The matrix patch was then sutured to the periphery, followed by incision closure after placement of small suction drains. Only one of the patients had device erosion and infection requiring system extraction. The matrix used in this study was AlloDerm® (Lifecell, Branchburg, NJ). This is an FDA-approved, acellular dermal matrix derived from human cadaveric skin and has several surgical applications.

Another acellular matrix product specifically designed for CIED, conforming to the shape and size of various CIED pulse generators, the CanGaroo® (Aziyo Biologics, Inc., Roswell, GA) may also serve in this role. The collagen matrix is derived from porcine small intestinal submucosa and has been shown to decrease inflammation and stimulate the ingrowth of healthy native tissue.

The advantage of using the acellular matrix products is the ability to deploy currently used devices with minimal additional surgical training, dissection, and increase in procedural time.

There is a significant biologic rationale for the use of acellular matrix products for reinforcing the thin patient CIED pockets. Anecdotal and early research experience is also promising; however, more experience and scientific data are needed before use of extracellular matrix to reinforce the device pockets of thin patients can be routinely prescribed.

## Conclusions

Elderly and emaciated patients with deficient subcutaneous fat present a unique challenge with respect to CIED pulse generator implant. Apart from poor cosmetic outcome from device protrusion through the skin, skin erosion and device/lead exposure necessitate device extraction. Various steps at the time of device implant planning and procedure can be taken to avoid this complication. The emergence of acellular matrix presents a promising new development in this direction.
